# The Impact of Gender Stereotypes on the Self-Concept of Female Students in STEM Subjects with an Under-Representation of Females

**DOI:** 10.3389/fpsyg.2017.00703

**Published:** 2017-05-17

**Authors:** Bernhard Ertl, Silke Luttenberger, Manuela Paechter

**Affiliations:** ^1^Department for Education, Universität der Bundeswehr MünchenNeubiberg, Germany; ^2^Federal Centre for Professionalization in Education Research, University of Teacher Education StyriaGraz, Austria; ^3^Educational Psychology Unit, Department of Psychology, University of GrazGraz, Austria

**Keywords:** female STEM students, impacts, self-concept, stereotypes, support

## Abstract

It's possible to assume that women who study STEM topics with a low proportion of females have successfully overcome barriers in school and the family, making them less prone to stereotypic views, and influences. The present study focuses on these kinds of factors and analyzes to which degree family factors, school-related factors, and individual stereotypes may influence a woman's academic self-concept. The following study presents a latent regression model which is based on a survey of 296 women from different German universities, all of whom are part of STEM programs of study that have <30% females. It was investigated to which degree individual stereotypes, support in school, and family support contribute to the self-concept in STEM. Gender stereotypes were negatively related to students' STEM-specific self-concept in the selected sample. This study also reveals negative family-related influences that lower a woman's self-concept. Positive predictors on the other hand included school aspects that are found in the students' favorite subjects at school. The results of the study provide important aspects for STEM education. Even though the students participating in the study presumably had good grades in STEM, stereotypes still corrupted their self-concept. One of the reasons for this might lie in stereotypes that attribute girls' achievements to diligence instead of talent. The results also point out that direct support, particularly by parents, can have a negative impact on female students' self-concept. Activities that are meant to support pupils directly may actually backfire and transport stereotypes instead. This stresses the need for indirect support during socialization, e.g., by providing opportunities for children to have positive experiences or by giving them the chance to meet role models that are enthusiastic about their STEM professions. These kinds of measures have the potential to spur students' interest in STEM subjects—something that in the present study proved to be especially beneficial for women's positive self-concept when studying STEM topics.

## Introduction

In most European countries, the proportion of females pursuing a career in STEM (Science, Technology, Engineering, Mathematics) is still alarmingly low. This holds especially true for occupations in technology and engineering (Blickenstaff, 2005; Ihsen, 2009; European Commission, 2015). The past decades have seen the proportion of females in these fields remain constant at approximately 25% in the EU, and even lower in Germany with approximately 18% [CEWS (Center of Excellence Women Science)., 2014]. One of the reasons females avoid STEM subjects lies in the negative and stereotyped perception(s) of these subjects (see Engeser et al., 2008; Schuster and Martiny, 2017). Stereotypical assessments here include expectations e.g., about a particular gender, as well as the attributions of abilities in specific domains. Such assessments are embedded in a broader cultural context of the individual (see Good et al., 2008). According to Bronfenbrenner's (1977) ecological systems theory, a major source of stereotypes lies within an individual's macro system, i.e., the cultural and social context of a person's societal group. The macro system refers to the overall values and customs that characterize a given social group which provide a framework for the interactions between the individual and its social context, e.g., the teachers at school or the family. Depending on the macro system and its values, stereotypes about professions, or subjects may vary among nations or cultures (see Nosek et al., 2009; Else-Quest et al., 2010). Many females in the Western world still believe the stereotype that professions and subjects in STEM are “male” domains (Nosek et al., 2009) and they often apply these kinds of stereotypes to the assessment of their own abilities in STEM (see e.g., Dresel et al., 2007).

Stereotypical classifications of professions and subjects have strong implications for females. They impair learning and prevent females from fulfilling their full potential. Stereotypes lower one's self-assessment and sense of competence, i.e., a person's self-concept (Marsh and Scalas, 2011). They even have an impact on career choices (e.g., Engeser et al., 2008; Schuster and Martiny, 2017).

Against this background, the present study investigates how stereotypes may explain female university students' self-concept in STEM. In this context, it is important to have a closer look into the different STEM subjects. Even if the term is used internationally, there are particularly differences about the definition of the science part. The German equivalent to STEM focuses only on “natural” sciences like physics, chemistry, biology etc., (see Ihsen, 2009). The English-speaking community also includes life sciences like medicine (e.g., European Commission, 2015; Eccles and Wang, 2016), while some authors, primarily from the US context also include social sciences in this definition (e.g., Su and Rounds, 2015). It is important to acknowledge this fuzziness when interpreting results with respect to STEM, because all these definitions, comprise subjects with a very low proportion of females, e.g., engineering as well as with a superior proportion of females like e.g., life sciences (see e.g., European Commission, 2015; Su and Rounds, 2015)—even if the proportions of females vary between the countries. This study focusses on a special group of female STEM students for reducing ambiguity: those who study a subject with an especially low proportion of females. We will label these STEM subjects having an under-representation of females as STEM-LPF (STEM subjects with a *l*ow *p*roportion of *f* emales). Studies with an especially low proportion of females have less than 30% (Buchmann et al., 2002). This means that for every female, more than two males study this subject. This group of female STEM-LPF students was selected because it could be expected that they are less prone to stereotypes after they have chosen what can be seen as a less-than-typical career path.

## Academic self-concept

An academic self-concept comprises a person's self-assessments in academic domains. It is formed through experience and interpretations of one's environment as it regards feelings of self-confidence, competence, and ability. It's influenced by evaluations of significant others, reinforcements, and attributions of one's own behavior (Marsh and Scalas, 2011). Such self-assessments may belong to two frames of reference (Rost et al., 2005): The external frame of reference is guided by a social comparison of one's own achievements with those of peers. The internal frame of reference is guided by a comparison within the individual, for example a comparison of abilities in various subjects. Students compare their achievement in one subject (e.g., mathematics) with their achievement in another (e.g., English).

The academic self-concept in a specific domain does not necessarily accurately reflect achievements. In a study by Ludwig (2010), female middle school students were much more critical of their abilities in STEM than male students even if they had the same grades. Similar results were found in the PISA studies (OECD, 2015). The academic self-concept of females who perform on the same level as their male counterparts in the PISA science scores was about one quarter standard deviation lower (OECD, 2015, p. 75). In most participating countries, females had a more critical academic self-concept in STEM than males. These kinds of differences can be downright vicious because research postulates reciprocal effects between the academic self-concept and achievements (see Marsh and Scalas, 2011). In their *reciprocal effects model*, pathways were found between students' achievements and their academic self-concept and vice versa. This means that, considering students on the same level of achievements, the students with the higher academic self-concept will advance in their achievements over the course of time while the others will lag. This effect may be explained by expectancy-value theory in how students with a higher academic self-concept in a domain have higher expectations regarding their chances for successful outcomes and as a result have a higher motivation to invest time and effort into learning activities in this domain (see Eccles et al., 1983; Eccles and Wang, 2016).

Attributions for causes of achievement also essentially contribute to the development of an individual's self-concept (see Möller and Köller, 1996). Successful achievements may be attributed to ability and thus enhance a positive self-concept, or they may be attributed to luck and have detrimental effects on the self-concept as a result (see Heider, 1958). Attributions are also related to learning motivation: Attributing academic failure to a lack of effort may increase effort for the next examination, while attributing failure to the lack of ability may cause resignation. Thus, the academic self-concept influences to which degree a student makes full use of her/his academic potential (see Jahnke-Klein, 2006). Studies show that female and male students differ in their attribution patterns in STEM fields (Beermann et al., 1992; Jurik et al., 2013). In comparison to males, although females seldom attribute success in STEM fields to ability, they do in fact attribute failure mostly to the lack thereof (Dickhäuser and Meyer, 2006). These kinds of dysfunctional attribution patterns interfere with the development of a positive self-concept and impair learning motivation (see also Ziegler, 2002; Dresel et al., 2007). All in all, a too-critical self-concept is an important reason why females believe they have inferior skills in STEM fields (see Wang et al., 2015; Eccles and Wang, 2016); why they are less motivated; and why they seldom consider a career in a STEM field at all (OECD, 2015).

School and family are two distinct environments that support the development of a student's academic self-concept. Different characteristics of classroom teaching show substantial effects on students' academic self-concept and their interest in a subject (Lazarides and Ittel, 2012). Comparisons in the classroom set an external frame of reference for the self-assessment and attribution of achievements (see Rost et al., 2005). Teachers' support in the attribution of achievements (Heller and Ziegler, 1996) can help students overcome gender-specific attribution patterns (Dresel et al., 2007). So teacher behavior can support students' interest and their development of a positive academic self-concept and encourage students to perhaps even experience STEM as their favorite field, all while keeping in mind that opposite effects are possible as well.

Within the family context, there is no in-class comparison. Here, parents' attributional beliefs serve as a frame of reference for a student's self-assessment (Viljaranta et al., 2015). Parents' beliefs about their child's ability have strong impacts on his/her self-assessment of ability (Tiedemann, 2000) and academic self-concept as a result. This makes parent support an important aspect in the context of STEM (Adya and Kaiser, 2005). However, if parents consider their child as being less capable, they may provide intrusive support with detrimental effects on the child's self-assessment (Pomerantz and Eaton, 2001). In other words: parents' influence on their children's academic self-concept can be ambiguous depending on their specific behavior, making it important that students experience support for their self-assessments at *both* school and at home (Adya and Kaiser, 2005). Of note here is that the effects of this support are subject to the particular support behavior. In the context of the STEM subjects, gender stereotypes can be seen as one reason why support measures may achieve the opposite effect.

## Stereotypes and their impact in STEM

The development of the academic self-concept begins in infancy and unfolds its most significant impact(s) after primary school (Senler and Sungur, 2009). Parents' and teachers' expectations and attributions of abilities and achievements essentially shape a child's self-concept (Dresel et al., 2007; Ludwig, 2010). They do not necessarily rely on objective assessments; often, parents underlie stereotypical evaluations which do not correspond to their children's actual achievements. For example, parents tend to regard daughters as being less talented in mathematics and science and reinforce dysfunctional attribution patterns as a result (Dresel et al., 2007).

### Explicit stereotypes as a threat to performance

Several studies on stereotypes have coined the term “stereotype threat” (Martignon, 2010, p. 221; Shapiro and Williams, 2012). In these studies, participants usually were confronted with a stereotype about a target group, e.g., females or members of a specific ethnic group. In the context of STEM, stereotypes would include males being more talented and successful in math and science. After confrontation with the stereotype, study participants worked on a task that is associated with the stereotype (Martignon, 2010, p. 221), and performance was compared to another group working on the same task that was not confronted with the stereotype. In nearly all studies on stereotype threat, females achieved worse results with mathematical tasks, and their interest decreased when they were confronted with the stereotype that women are less talented in mathematics (Shapiro and Williams, 2012).

Owens and Massey (2011) describe two mechanisms that explain why stereotype threat occurs. The first mechanism works via internalized stereotypes; this means the person has internalized the stereotype and identifies him/herself with the target group. Consequently, he/she invests less effort in the task and the stereotype threat becomes a self-fulfilling prophecy. The internalization of the stereotype also has a negative effect on the academic self-concept (Heckhausen, 1989) and is accompanied by a reduction in motivation and effort (Möller and Köller, 1996). The second mechanism works via external stereotypes (Owens and Massey, 2011). In this case, the person does not necessarily identify him/herself with the stereotype, nor does he/she need to believe the stereotype. Confrontation with the stereotype, however, affects the perception of task difficulty, increasing strain and tension. Rumination about the stereotype uses up resources that are otherwise needed for task completion, impairing performance as a result (see Macher et al., 2015). This research shows that even females who believe themselves to be competent and pursue a career in STEM still can be impaired by stereotype threat.

### Influence of stereotypes communicated by significant others

Stereotypes are also communicated by significant others such as parents or teachers (Gunderson et al., 2012). Tiedemann (2000) showed in his study on pupils in primary school that mothers as well as teachers based their feedback on children's competence in mathematics not only regarding previous grades but the respective child's gender as well. Mothers were even more prone toward gender stereotypes than teachers. Stereotypes were especially strong in feedback on achievements and had a significant impact on the children's self-concept (Tiedemann, 2000). In a study by Kiefer and Shih (2006), students were especially receptive to teacher feedback that was associated with gender stereotypes. According to Dickhäuser and Meyer (2006), girls mainly rely on perceived teacher evaluations of their ability when making math ability assessments and thus are very susceptible to incorporating significant others' stereotyped evaluations into their own self-concept (see also Xu, 2016).

Parents' and teachers' gender stereotypes manifest themselves not only in communication, but in dysfunctional support for their children or students as well. When parents endorse specific gender stereotypes (e.g., boys are better in STEM, girls are better in languages), they are more likely to uninvitedly intrude on homework, undermining children's confidence in these areas, and weakening their self-concept (Bhanot and Jovanovic, 2005). These kinds of long-term influences by parents and teachers may have a significant influence over the years not only on motivation and achievement but regarding career choices as well (Bleeker and Jacobs, 2004).

## Research question

The academic self-concept is a key variable in explaining learning and motivation in specific academic domains. It is also of interest in explaining career choices and perseverance in a specific profession. However, it does not always rely on “objective” data such as actual achievements, but is instead subject to distorting influences such as internalized stereotypes as well as external stereotypical attributions by others.

The present article looks more closely into the academic self-concept of a special group of females: university students in a STEM-LPF subject with a notable underrepresentation of women (equal to or less than 30% females). It can be expected that these females would tend to be confident regarding their academic self-assessments in STEM fields, and less prone to stereotypical attributions concerning females' lack of abilities here. Therefore, the research question will investigate:

To what degree do STEM-LPF students' own stereotypes in comparison to school- and family- related factors contribute to their academic self-concept in STEM?

Regarding this research question, we would still expect a negative effect of stereotypes. However, due to a lack of research in the field, we cannot provide hypotheses about its strength within the context of the ambiguous effects of school and family factors.

## Method

The focus of this paper is primarily on a quantitative study with 296 female STEM-LPF students. For strengthening these results, we will also provide evidence from a qualitative study with STEM students that took part in an earlier stage of the project. Students of the qualitative study were also invited to participate in the quantitative one but as this was an anonymous survey there was no control of participation.

### Quantitative study

The sample employed in the quantitative study is part of a larger sample that was gathered in the EU research project SESTEM in six European countries. Five hundred and sixty seven female university students in STEM fields participated in Germany. Ertl et al. (2014) analyze the entire German sample (including students in STEM areas without female underrepresentation) with a focus on motivation and the academic self-concept.

#### Participants

The present study focuses on a sub-sample of 296 female STEM-LPF students: females who studied one of these STEM subjects that have a proportion of equal to or lower than 30% females. This sample includes 296 students in subjects including mechanical engineering (*n* = 97), computer sciences (*n* = 48), physics (*n* = 39), metal engineering (*n* = 36), civil engineering (*n* = 34), electrical engineering (*n* = 32), and other STEM subjects (*n* = 10).

#### Measures

A specific questionnaire was developed for the study. Items were deducted from theory and adapted for the field of the study. During this process, all six partners of the SESTEM project consortium brought in aspects within their field of expertise. Seeking and including expert judgment on the content of a questionnaire, on item formats, item contents, and scoring systems enhance content validity of a measurement instrument. Then, the consortium negotiated about the inclusion of the different scales weighting between satisfying the needs of the different partners, adopting existing scales, and keeping the questionnaire as short as possible for maintaining students' motivation for answering the questions. This resulted in a final questionnaire in an English language version, which was translated into further five national languages including German. These six language versions were implemented as a LimeSurvey multi language questionnaire. The students reported in this paper answered the German language version. They were asked about:

1. Their *majors* or the subject combination they had chosen for their degree. Based on the data from the German Federal Statistical Office [Destatis (Statistisches Bundesamt), 2013], majors were classified with respect to the proportion of females.
2. Their *parents' professions*. These were classified according to whether they were from the field of STEM (coded as STEM/not STEM).
3. Their *academic self-concept in STEM* on a five-point Likert scale (4 items, see Table 1). Higher values indicate a more positive self-concept.
4. Their internalization of gender *stereotypes* was measured by three scales: interests (7 items), abilities (5 items), and conformance (2 items). Each of these scales was based on a five-point Likert scale (see Table 1). Higher values indicate stronger stereotypes.
5. *School factors*. Here the following variables/scales were measured: First, a score was derived from students' STEM favorites (derived from students' three most favorite subjects at school. Subjects from the field of STEM that are known for association as a “male domain” were summed up to a score. This means that the score includes subjects such as mathematics, physics, or computer sciences, but not subjects like biology). Higher values indicate more favorite STEM subjects. Second, STEM support in school was operationalized by teachers' and school activities that facilitated the interest in STEM (e.g., “Were there activities in secondary school that encouraged your interest in STEM?” These answers were also summed up and mapped onto a range between 0 and 5) with higher values indicating more support. Third, a five-point Likert scale regarding students' perception of teachers' stereotyped behavior (4 items, see Table 1).
6. *Family factors* with respect to family support. This was surveyed by different areas in which students may have received support and the persons that supported the students (e.g., “Who supported you in mathematics: father/mother?”) Answers were distinguished with respect to the supporting person and the supported field and summarized into a score for support by parents generally, as well as for support in specific areas (mathematics/science). These scores were mapped regarding their theoretical maxima and minima on a range between 0 and 1. Altogether three variables were derived: Parents' support in math, parents' support in STEM, and parents' general support. Higher values indicate stronger support.

**Table 1:** **Overview on the scales used for the study with the number of items, an exemplary item, and the internal consistency**.

**Scale**	**Items**	**Exemplary item**	**Cronbach's α**
Academic self-concept STEM	4	“I am not skilled enough in mathematics for choosing a career in STEM”	0.82
Stereotypes about interests	7	“Girls show less interest in STEM subjects than boys”	0.73
Stereotypes about ability	5	“Girls have lower skills in STEM subjects than boys”	0.70
Stereotypes about conformance	2	“Females that are working in the field of STEM have to be like men”	0.77
Stereotyped teacher behavior	4	“Teachers are more likely to encourage boys to take STEM subjects”	0.88

*According to Paechter et al. (2013), Cronbach's α with .70 and more can be considered a satisfying indicator of the internal consistency of a scale. Data for academic self-concepts were recoded so that higher values indicate a higher self-concept. For stereotype variables, higher values mean higher stereotypes*.

Table 1 gives an overview of the different Likert scales including the number of items, an exemplary item, and the internal consistency of the scale. The reported consistency measures relate to the whole sample of 567 students. Missing items of single scales were imputed; missing scales were treated as missing. Table 2 provides an overview of all scales including their value range, their means, and their standard deviations.

**Table 2:** **Ranges, means, and standard deviations for the reported scales**.

	**Range**	**Mean**	**Standard deviation**
Academic self-concept STEM	1–5	4.58	0.55
**STEREOTYPES ABOUT**			
– Interests	1–5	3.14	0.67
– Ability	1–5	2.20	0.63
– Conformance	1–5	1.64	0.86
**SCHOOL FACTORS**			
– STEM favorites	0–3	1.54	0.75
– School support	0–5	2.33	2.07
– Stereotyped teacher behavior	1–5	2.51	0.91
**FAMILY FACTORS**			
– Mathematics support	0–1	0.15	0.20
– STEM support	0–1	0.14	0.20
– Parent general support	0–1	0.36	0.20

### Qualitative study

The quantitative study was complemented by a qualitative study. It comprised interviews based on a semi-structured interview protocol (for the complete set-up of the qualitative studies see Mok and Ertl, 2011). Interviewees were contacted by personal contact, email, and via STEM-related distribution lists. A sample of 11 female students of STEM subjects like mathematics, physics, engineering, and STEM-related teacher training from three different universities participated in the qualitative study; five students studied a LPF subject (civil engineering *n* = 2, physics *n* = 3).

## Results

In the following, we will first report results of the quantitative study. The results section will first provide insights into the descriptive outcomes. Then it will describe the results of the confirmatory factor analysis for the factors of stereotypes, school, and family. It will finally present a structural equation model that provides insights into the impacts of each of the factors onto the students' academic self-concept in STEM and illustrate these afterwards by the interviews with these five students of the qualitative study.

### Descriptive statistics

Of the 296 students, nearly the half of the students (139) had a father working in a STEM profession, while more than 10% (31) had a mother in STEM.

Most students showed a very positive self-concept (*M* = 4.58; the means described in the following relate to a scale of 1–5, with 1 as the lowest and 5 as the highest value). We could find distinctive occurrences with respect to the internalization of stereotypes between the students. The students agreed mostly that girls and boys have different interests (*M* = 3.14). They agreed less about stereotypes regarding a stereotype distribution of abilities (*M* = 2.20), and least of all about the need for conformance (*M* = 1.64; see Table 2).

With respect to school factors, 26 students had three favorite subjects from STEM at school, 129 students two, 121 just one, while 20 had favorite non-STEM subjects (*M* = 1.54). They received a moderate amount of STEM support in school (*M* = 2.55 of a maximum of 5), and also perceived a moderate amount of stereotyped teacher behavior (*M* = 2.51 of a maximum of 5).

Considering family factors, the amount of parents' support in math (*M* = 0.15 of a maximum of 1) and STEM (*M* = 0.14) was low. General support by the parents was low to medium (*M* = 0.36).

To analyse the distribution of the data, we used the values of the skewness and kurtosis. West et al. (1995) set the criteria for indicators used in structural equation models at a value >2 for skewness and >7 for kurtosis for deviation from normal distribution. All scales meet the requirement of normal distribution.

### Latent regression analysis

Latent regression analysis was used to test relationships between the variables in a multivariate, multiple regression context. Structural relationships between multiple dependent variables and multiple independent variables can be analyzed simultaneously. Regression analyses are specified at the latent level and are corrected for measurement error at the level of the independent and dependent variables. Latent regression analysis has the advantage that the relationship between variables in the regression model can be estimated more accurately. At least two manifest variables (or indicators) are required for each latent variable (factors) in a latent regression model (Geiser, 2013). The data were analyzed with Mplus 6 using a maximum likelihood estimator. The goodness of fit of the data to the hypothesized model was assessed using the following indices: χ^2^/df, comparative fit index (CFI), root mean square error of approximation (RMSEA), and standardized root mean square residual (SRMR).

The model fit indices suggest a good fit of the latent regression analysis model (χ^2^/df = 1.422; CFI = 0.979; RMSEA = 0.038; SRMR = 0.049). Generally, values of χ^2^/df <2, CFI > 0.95, RMSEA < 0.05, and SRMR < 0.05 are considered as indicators of good model fit (Papousek et al., 2012).

Table 3 displays the standardized solutions for the latent regression analysis with three the factors of stereotypes, school, and family. Each factor comprises different variables that describe stereotypes rooted in the culture or encountered in school or the family.

**Table 3:** **Standardized coefficients for the latent regression analysis**.

**Indicators**	**Factors**	**β**	***S.E*.**	***p***
Stereotypes about interests		0.274	0.115	0.017
Stereotypes about ability	Stereotypes	0.590	0.115	0.000
Stereotypes about conformance		0.379	0.085	0.000
STEM favorites		0.614	0.134	0.000
School support	School	−0.326	0.087	0.000
Stereotyped teacher behavior		−0.274	0.098	0.005
Mathematics support		0.784	0.032	0.000
STEM support	Family	0.806	0.031	0.000
Parents support		0.787	0.032	0.000

The model shows that the three indicators of stereotypes about interests (β = 0.274), stereotypes about ability (β = 0.590), and stereotypes about conformance (β = 0.379) are positively related to the factor stereotypes. Three indicators are related to the factor school: STEM favorites in school (β = 0.614), school support (β = −0.326), and stereotyped teacher behavior (β = −0.274). The three indicators support in mathematics (β = 0.784), support in STEM (β = 0.806), and support by parents (β = 0.787) are high positively related to the latent factor family.

**Figure 1:**
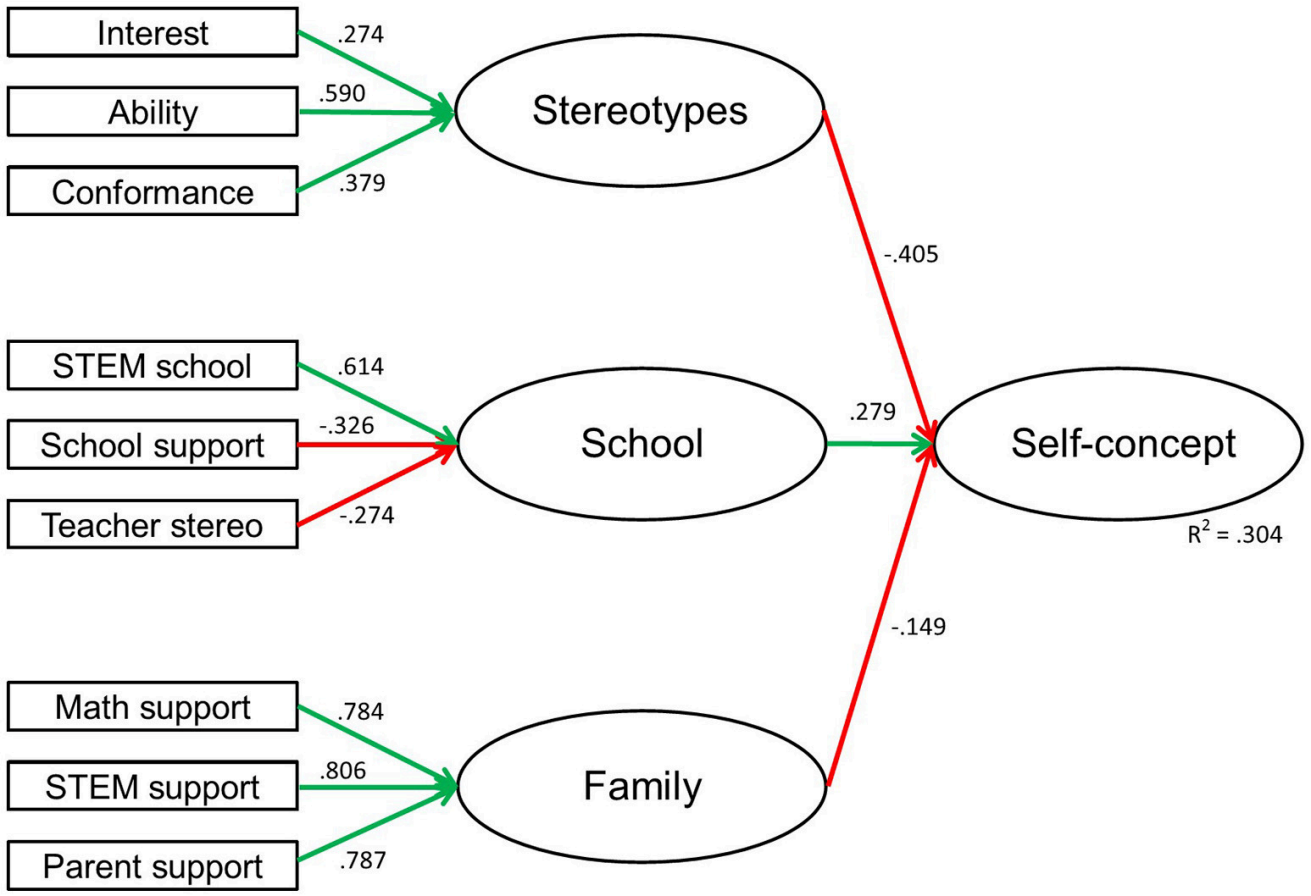
**Latent regression analysis self-concept**.

The regression coefficients between the three factors stereotypes, school, and family and self-concept in STEM of students show the following result: Students with higher levels of experienced stereotypes (e.g., females have fewer skills or interest in STEM subjects, females in STEM have to be like men) report lower self-concepts in STEM domains (β = −0.405). The model shows a moderate relationship between the latent factor school and students' self-concept (β = 0.279). Students who reported a higher number of favorite STEM subjects in school have a higher self-concept whereas higher levels of school support and teachers' stereotypes indicate a lower and less positive self-concept in STEM. There was a weak relationship between the latent factor family and the self-concept of students (β = −0.149, *p* = 0.053). A higher level of support (math, STEM, parents) indicates a lower self-concept. The total variance of self-concept that can be explained by the factors is *R*^2^ = 0.304. Figure 1 gives an overview of indicators and factors of the latent regression analysis model.

Correlations between the three latent factors were allowed in the model specification. We found low to moderate, but non-significant correlations between the three latent factors.

### Evidence from the qualitative study

The analysis of the qualitative study aims to illustrate the latent variables of the quantitative one. Students' statements can give evidence for the latent factors of the quantitative study with respect to the impact of stereotypes and family. School factors were just mentioned in a few words, e.g., that students had taken advanced courses in mathematics (I57) or physics (I30, I57) or that they had enjoyed mathematics in school (I54). We will present the English translation of the statements; the German original version can be found in the project report (Mok and Ertl, 2011).

#### Impact of stereotypes

With respect to the impact of stereotypes, students mentioned that they were taking an untypical career path and that their social environment was surprised by this kind of career choice. A civil engineering student mentioned that surprise with respect to her friends: “*They were quite surprised,”* I54, L.99. She further elaborated this untypical career with respect to the lack of acceptance of women in the construction area: “*The problems are bigger for women [in STEM] e.g., to be accepted in particular in the construction domain. There you need particularly technical knowledge and you have to know how to behave,”* I54, L.124ff. This aspect was also emphasized by I1: “*As a woman you'll be seen different in a technical profession,”* I1, L.16f. These untypical career choices also result in a perceived lack of role models and contact persons, e.g., female professors (“*There are few female professors,”* I30, L.69). Thus, also the interview data highlights that students are aware that they are studying an untypical subject and name surprise of their friends about their study choice, obstacles for working in the untypical field, as well as missing role models.

*Family impact*. With respect to family impact, all students mentioned either that their father (I1, I54, I57) and/or mother (I1, I54) is in a STEM profession *(“Both of my parents are teachers but my father has also studied physics and got a diploma […],”* I57, L.47f.)—or that their parents supported their specific interest in STEM, e.g., by books (I35) or electronic construction toys (“*That my parents had already impacts on me because I also had got electronics experiments kits as a child,”* I30, L.19f.). Most parents, particularly those in a STEM field, encouraged their daughters' pursuing a STEM career: “*The parents enhance the STEM-career because they are working in this field themselves”*(I1, L.41). Some students further elaborated their parents' pleasure at their daughters' career wish “*My father was happy for me and my mother too.”*(I57, L.59).

Parents also supported their children in case of difficulties, e.g., with homework (“[…] I had the opportunity to ask my father of course if I had e.g., pretty problems in mathematics or physics and he was able to help me,” I54, L.28ff.) or by providing stimulating tasks (“My father had written a computer program that provided us arithmetic problems when we attended primary school,” I57, L.36f.).

Yet, some students also described that their parents were doubtful about their ability for pursuing a STEM career (“My dad told me afterwards that he hadn't thought that this is the right thing for me […] because I have an already an understanding for logical relations but I have not an all-embracing one,” I54, L.105ff.) or that they questioned their decision (“my father appreciated my decision but my mother mentioned—although she was also working in the STEM field herself—that I should really think about my decision.” I35, L.56f.).

The results of the interviews stress the ambiguity of the family factor: Firstly, all parents had a STEM-affine background. They could provide content-specific support and foster their daughters' cognitive development in STEM. However, such support may also evoke an attribution of lower abilities in STEM. For example, one participant first mentioned that her father was very helpful when dealing with problems in STEM—but later she described how her father didn't trust her the ability for pursuing a STEM career. Thus, parents' support may be connected to implicit assumptions about their daughters' ability and these assumptions may influence their daughters' academic self-concept in STEM.

## Discussion

The results of the quantitative study were able to show that the model presented is appropriate for explaining students' self-concept. This is indicated by the good model fit indices, as well as by the amount of explained variance: The model explains 30.4% of the total variance of students' self-concept, which is nearly a third of the variance. Results of the qualitative study could furthermore give insights how to interpret the effects of the latent variables. In the following, we will discuss the relationships between stereotypes, school, and family factors, the self-concept, as well as the limitations of the study.

### Relationships between stereotypes, school, and family factors, and the self-concept

All three facets of stereotypes (stereotypes about females' abilities, interests, and need for conformance) contributed negatively to the academic self-concept. Remarkably, stereotypes regarding females' abilities in STEM subjects were most strongly related to their self-concept. This is particularly important because the females of this study were already studying a so-called “male” STEM-LPF subject. The descriptive data showed that even these students share stereotypes, indicating that stereotypes even affect students who are already enrolled in a very gender-untypical course of study. Stereotypes about a need for conformance in the work environment and the different interests of females and males also contributed to the factor stereotypes. Also, result from the qualitative study indicate that there is a special need to behave in the domain. This result is of particular interest because it means that the STEM-LPF students acknowledge different interests of females and males, while they at the same time see the context of the “male” work environment and the need for showing conformance. They appear to use conformance to the work environment as a part of their identity construction (see Kessels and Hannover, 2004, p. 400). This may also be an aspect of identity bifurcation (see Pronin et al., 2004) in how females in these subjects disavow some of their own characteristics that are, stereotypically, negatively associated with success in STEM careers.

In contrast, the three indicators of the latent factor school differ in their contribution. Students' favorite subjects in school, which could be seen as an indicator of their interest in STEM, or beneficial role modeling by teachers, were positively related to the self-concept. This stresses the importance of school factors for career choice. These may relate to interesting and gender-sensitive classes (Faulstich-Wieland et al., 2008; Ertl and Helling, 2011), role modeling (Kessels and Hannover, 2008), and providing appropriate attribution patterns (Dresel et al., 2007). However, specific support at school and teachers' stereotypes had a negative relationship with the factors of school and self-concept. Teacher stereotypes, e.g., teachers encouraging boys to choose STEM subjects more strongly than girls, can be seen as a specific occurrence of the stereotype threat with the respective consequences (e.g., Good et al., 2008; Owens and Massey, 2011). It's fairly obvious that these kinds of actions provide a counterpart to students' interests in STEM. In contrast, teachers supporting their female students have the intention that they make further progress in STEM subjects. Nevertheless, these activities may in fact run counter to their interests in STEM, which may be the result of different reasons: The first aspect relates to the development of the self-concept in STEM. If students receive special support in STEM, they may interpret this action as a compensation for their lacking ability and therefore reduce their self-concept (Pomerantz and Eaton, 2001). This is certainly the case when students receive intrusive support (see Bhanot and Jovanovic, 2005). From this line of argumentation, it is essential to investigate methods and implementations of support that are not detrimental to a students' self-concept. This result might also be explained by the “doing gender” approach: When giving specific support to females in STEM, their gender will be overemphasized, evoking a stronger identification with the stereotyped group of females in STEM (see Faulstich-Wieland et al., 2008). What this means is that supporting activities may in fact unfold their detrimental effects via two different mechanisms: one by giving supported students the message that their individual ability is not sufficient enough to succeed without support; and the other by overemphasizing their affiliation to a stereotyped target group.

Family factors were negatively related to the students' self-concepts, i.e., they impair a positive self-concept. This factor consisted of support by the parents and support in mathematics and STEM. Notably, all three aspects showed rather dysfunctional effects. With respect to family factors, the qualitative study could provide a several hints for interpretation. All students mentioned that their parents were very helpful and supportive. However, one student explicitly mentioned her father attributing her as not gifted enough for a STEM career while giving her support. This is in line with research about intrusive support patterns that are detrimental to a student's self-concept (see Bhanot and Jovanovic, 2005). Furthermore, one student reported her mother encouraging her to re-think her career decision for STEM which stresses the impact of significant others in career decisions (see also Xu, 2016).

The results generally suggest that the school environment provides more positive impacts than the family. This may relate to the different attribution patterns of teachers and parents (Dresel et al., 2007). Teachers can provide much better attribution patterns in the context of the reference frame of a class's performance than parents who are primarily focused on their child with their beliefs as the key frame of reference. This stresses the need to focus on both school as well as on home environments as essential factors in facilitating students' self-concept (see also Eccles and Wang, 2016).

### Limitations

A strength of this study lies in the more ecological approach as foreseen in the Bronfenbrenner (1977) model. This approach provided more insights into stereotypes as well as interactions at school and at home. It at the same time included a major challenge for research that relates to the issue of how the study variables were self-reported by the students, with some of the variables even being reported retrospectively. It would have been desirable to research these issues in a longitudinal design in an effort to achieve greater insight into causal relations and the development process of stereotypes, interests, achievements, and the individuals' self-concepts. However, such a design would raise the issue of the necessary sample size at the primary school level to gain the respective number of students at the university level. A further aspect relates to the implementation of the Bronfenbrenner (1977) model in the latent regression analysis. Here, it would have been desirable to provide more interactions between the different levels this model proposes, even though such an approach would also require a longitudinal study design. In contrast, our research can provide insights into different dimensions influencing a STEM-LPF student's self-concept.

## Implications

The results of the study provide important aspects for science education. Even though the students participating in the study almost certainly had good grades in STEM, stereotypes still corrupted their self-concept. One of the reasons for this might lie in stereotypes that attribute achievements of girls to diligence instead of talent (see Kessels, 2015). STEM subjects, particularly these with a low proportion of females, are stereotyped as requiring an extremely high level of talent to succeed. Good grades, although they are seen as a prerequisite for a STEM-LPF course of study (see Ihsen, 2009), are not sufficient to support a self-concept necessary for females to choose STEM-LPF subjects. This means that even students with good grades need support in developing efficient attributes for success (Ziegler, 2002; Dresel et al., 2007). This may be implemented e.g., via support for a student's decision about what to study (see Ertl et al., 2014). This kind of support provides the implicit attribution pattern that a female student is “gifted enough” to study a male-associated STEM subject (see Dresel et al., 2007) and could thereby be seen as a specific method for strengthening an individual's self-concept. It can also be seen from a systemic point of view as an example of appropriate role modeling when it opens perspectives for identification with a subject or with a professional within a subject (see Hannover and Kessels, 2004).

A further aspect relates to interests at school. These may positively influence students' self-concepts and career choices if they have the chance to recognize a STEM subject as their favorite. This stresses the need for gender-sensitive teaching and a careful attention to gender-specific group processes in the classroom (see Ertl, 2010). Didactic measures that incite interest are, for example, hands-on activities that are oriented toward the students (see Paechter et al., 2006), or research clubs that allow students to obtain actual experiences about STEM-LPF professions (see Prenzel et al., 2009). The results of the last PISA studies confirm these results and assumptions while pointing out the necessity to overcome gender gaps and support females' interest in STEM subjects (OECD, 2016).

Direct support, particularly by parents, had a negative impact in the present study. This result suggests that activities that are meant to support students directly may achieve the opposite effect and transport stereotypes instead (see e.g., Tiedemann, 2000). This stresses the need for indirect support during socialization, e.g., by providing opportunities for children to have positive experiences (Sonnert, 2009) or by giving them the chance to meet role models who are enthusiastic about their STEM professions (see e.g., Mok and Ertl, 2011). One particular aspect of this may lie in the provision of mentoring programs (see Stein, 2013) that allow students to accompany their mentors over a longer period of time.

## Ethics statement

The study was performed in accordance with the 1964 Declaration of Helsinki and the American Psychological Association's Ethics Code. Review and approval was not required for this study in accordance with the national and institutional requirements. Participants gave consent to participate in the study at the beginning of the qualitative interviews and by submitting the online questionnaire for the quantitative study.

## Author contributions

All authors listed, have made substantial, direct and intellectual contribution to the work, and approved it for publication.

## Funding

Parts of this paper were funded by the EU (LLP-Program, Project SESTEM 505437-llp-2009-GR-KA1-KA1SCR).

### Conflict of interest statement

The authors declare that the research was conducted in the absence of any commercial or financial relationships that could be construed as a potential conflict of interest.
